# Trends in the incidence of adenocarcinoma of the oesophagus and cardia in the Netherlands 1989–2003

**DOI:** 10.1038/sj.bjc.6603798

**Published:** 2007-05-15

**Authors:** M van Blankenstein, C W N Looman, P D Siersema, E J Kuipers, J W W Coebergh

**Affiliations:** 1 Department of Gastroenterology and Hepatology, Erasmus MC, Erasmus University Medical Centre, Rotterdam, The Netherlands; 2 Department of Public Health, Erasmus MC, Erasmus University Medical Centre, Rotterdam, The Netherlands; 3 Comprehensive Cancer Centre South, Integraal Kankercentrum Zuid, Eindhoven, The Netherlands

**Keywords:** epidemiological trends, adenocarcinoma of the oesophagus, adenocarcinoma of the cardia, Barrett's oesophagus, age–period–cohort pattern analysis

## Abstract

Over the 15-year period 1989–2003, the incidence of oesophagus–cardia adenocarcinoma in the Netherlands rose annually by 2.6% for males and 1.2% for females. This was the net outcome of annual increases in the incidence of adenocarcinoma of the oesophagus (ACO) of 7.2% for males and 3.5% for females and annual declines in the incidence of adenocarcinoma of the gastric cardia (AGC) of more than 1% for both genders. Nonlinear cohort patterns were found in females with ACO and for both genders in AGC; a nonlinear period pattern was observed only in males with AGC. These differing epidemiological patterns for ACO and AGC do not support a common aetiology. Proposed underlying factors for the rise in ACO incidence appear to have little effect on AGC incidence. This and the secular decline in smoking among males may have led to the decline in AGC incidence.

Over the past 30 years, the incidence of adenocarcinoma of the oesophagus (ACO) has gradually exceeded that of squamous cell carcinoma (Heitmiller and Sharma, 1996).

The recognition that in the oesophagus, adenocarcinomas can (Morson and Belcher, 1952; Naef *et al*, 1975) and mainly do (Haggitt *et al*, 1978) develop in a columnar-lined (Barrett's) oesophagus has underlined the necessity of distinguishing between these adenocarcinomas (ACO) and the adenocarcinomas of the gastric cardia (AGC), which do not originate in the oesophagus. Where this distinction is not made, we employ the term oesophagus–cardia adenocarcinoma. A steady rise in the incidence rates of the oesophagus–cardia adenocarcinoma has been reported from the United States and Europe (Bollschweiler *et al*, 2001; El-Serag, 2002). However, whether this rise involved both ACO and AGC to the same extent has remained unclear, as most registries did not distinguish them. This is not surprising because, even for pathologists examining resected specimens, the distinction is often problematic (Siewert and Stein, 1998; Driessen *et al*, 2003). In Denmark, in the period 1989–1995, an AGC to ACO incidence ratio of 1.8–1 in males was reported (Eurocim, 2001); a panel of experts reviewing the original clinical data reversed this ratio to 1–2.4 (van Blankenstein *et al*, 2005). The Netherlands is among those countries that have the highest incidence rates of both ACO and AGC (Corley and Buffler, 2001).

By analysing the differential time trends in ACO and AGC incidence over a 15-year period and examining their age-cohort-period patterns, we have estimated their respective contributions to the rising incidence of the oesophagus–cardia adenocarcinoma in the Netherlands.

## MATERIALS AND METHODS

The Netherlands, a country of 16 million inhabitants, has nine Regional Cancer Registries, hosted by the regional Comprehensive Cancer Centres, to which both hospital medical record departments and pathology departments report all malignancies. The Netherlands Cancer Registry in turn collates these data. The ascertainment of symptomatic oesophageal cancer in the Netherlands is likely to be high, as it practically always results in endoscopic and histological diagnosis for curative or palliative interventions (Siersema *et al*, 2005).

For the 15-year period 1989–2003, the Netherlands Cancer Registry provided annual age- and gender-specific incidence data for ACO (the combined data for ICD.O regions C.15.3, C15.4, C15.5 and C15.9) and for AGC (ICD.O region C.16.0), both ICD.O morphology 8140/3 by 3-year age groups. This allowed estimation of time trends and age–period–cohort analysis for each cancer by log-linear Poisson regression models. Incidence rates were also provided as European standardised rates (ESR) by 5-year age groups. In addition, ACO incidence data by 5-year age groups were provided separately for each of the three levels of the oesophagus, upper-, middle- and lower-third and unspecified (ICD.O regions C.15.3, C15.4, C15.5 and C15.9).

For the cohort models, a mean year of birth was calculated for each 3-year age group. The estimated drift parameters constituted the annual percentage change in incidence, corrected for age and population size. We used linear splines to test for nonlinear period and cohort patterns. In any observed annual percentage change, the choice between a period and a cohort effect could only be made if one or the other spline was nonlinear. Therefore, instead of one exponential curve spanning the whole period, we extended the model to three exponential lines connected by knots at 1993 and 1998 for period estimates, and 1926 and 1944 for those of birth cohort. In both cases, the knots were placed at (approximately) one- and two-thirds of the time axis, without attempting to optimise this choice. Likelihood ratio tests (comparison of scaled deviances) indicated whether significant nonlinear period or cohort effects were present (Clayton and Schifflers, 1987a, 1987b). Male to female ratios were estimated from a model including incidence year, age group and gender.

## RESULTS

### Incidence (ESR) and trends by log-linear regression models

Over the 15-year period 1989–2003, ESR of oesophagus–cardia adenocarcinoma rose by 34% (from 7.4 × 10^5^ to 10.0 × 10^5^) in males, by 25% (from 1.7 × 10^5^ to 2.1 × 10^5^) in females and by 33% (from 9.1 × 10^5^ to 12.1 × 10^5^) for both combined.

**Figure 1:**
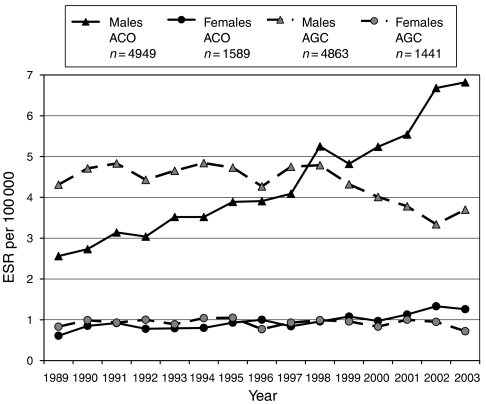
The annual European standardised rates for ACO and AGC by gender for the period 1989–2003.

The ESR and time trends for ACO and AGC by gender for these years are shown in Figure 1. The mean ESR of the two tumours over the total 15-year period was practically identical, for ACO, 4.3 × 10^5^ in men and 0.96 × 10^5^ in females and for AGC, 4.3 × 10^5^ in males and 0.92 × 10^5^ in females. Initially, male AGC incidence was far in excess of that of ACO. However, from 1998 onwards, the combination of steeply rising ACO incidence and a downward AGC trend reversed this relationship. A similar, but less pronounced, pattern was seen in females. The incidence trends, after correction for age, for both genders over the 15-year period 1989–2003 are shown in Table 1. Annual AGC incidence declined by −1.7% in males (*P*=0.0002) and –1.2% in females (*P*=0.05). In contrast, ACO incidence increased by 7.2% p.a. (*P*<0.001) for men but only 3.5% p.a. (*P*=0.006) for females. Table 1 also presents the trends in ACO incidence at three levels of the oesophagus. In males, these increases were: upper-third, 8.9% p.a.; middle-third, 6.5% p.a. and lower-third, 8.8% p.a. (*P*<0.0001 for each, with no significant differences between the three levels). In females, there were no significant annual increases at the upper- and middle-third, but, at the lower-third, an annual increase of 6.1% was found, *P*<0.0001. In 458 of 6538 cases of ACO (7%), no level was specified, with no discernable trends.

We did observe an unexpected similarity between both genders in the numbers of cases of ACO at the upper- and the middle-third. This was the result of a surge in the number of ACO cases in females over 75 years; upper-third: males 18 (ESR 0.44), females 53 (ESR 0.65); and middle-third: males 146 (ESR 2.6), females 206 (ESR 2.5).

The age- and gender-specific annual increases in ACO incidence are presented in Table 2. These increases were highest in the 40–60-year age group, decreasing significantly for both genders at ages 61–84 (*P*=0.03). However, in males this decrease amounted to only 25% against 62% in females. Consequently, the 1.2 : 1 male/female ratio at ages 40–60 rose to 2.3:1 at ages 61–84, finally resulting in annual rates of increase of 7.2% for males and 3.5% for females.

### Age–period–cohort models

The results of the cohort and period estimates are shown in Table 3. For each tumour, the mean changes in the annual incidence for each of three periods (before 1926, 1926–1944 and after 1944) were differentiated by year of birth for cohort effects and by incidence year for period effects. Significant differences between the values for the three periods indicated a nonlinear cohort or period effect.

There were no significant nonlinear cohort or period effects in males with ACO. In contrast, females with ACO showed a significant nonlinear cohort effect (*P*=0.006), with the greatest increase in the 1926–44 cohorts, but here again, there was no significant period effect (*P*=0.64).

**Figure 2:**
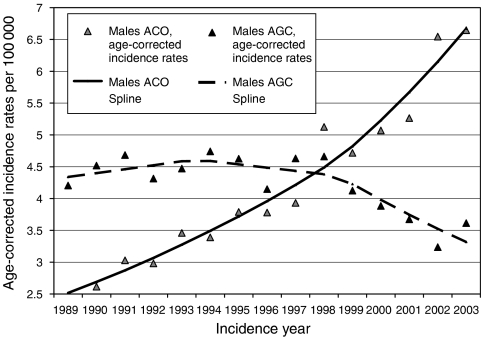
The age–period models for both ACO and AGC in males.

For AGC, a nonlinear cohort effect was found in males, *P*<0.0002, demonstrating a steadily declining trend in cohorts born after 1926. This was in contrast to the nonlinear cohort effect in females, *P*=0.01, which demonstrated a rising trend in cohorts born after World War II. Finally, for AGC a nonlinear period effect in males, *P*=0.0001 (Figure 2), partly mirrored in females, *P*=0.3, suggested a decline caused by a period effect setting in around 1995.

## DISCUSSION

These results from the Netherlands confirm the worldwide trend for increasing incidence of oesophagus–cardia adenocarcinoma (Bollschweiler *et al*, 2001; El-Serag, 2002). While in the Netherlands, this rising trend was very pronounced for ACO, the AGC incidence rates actually declined for both genders. Our major finding is that, at least in the Netherlands, the rising incidence of oesophagus–cardia adenocarcinoma over the past 15 years was entirely due to ACO, which, despite their clinical similarity, points to aetiological differences (Wijnhoven *et al*, 1999).

In the years 1968–1994, ACG incidence was already declining in the Netherlands (Laheij *et al*, 1999). However, where previously (ICD 9) visible Barrett's oesophagus was considered desirable for the diagnosis of ACO, current practice is to be guided by localisation of the major bulk of the tumour (Bytzer *et al*, 1999; Driessen *et al*, 2003). This revision could have resulted in a diagnostic shift from AGC to ACO, thereby explaining the period effect observed in the declining AGC incidence. However, in males this is contradicted on two grounds: (1) by the identical rising trends in the incidence of ACO localised in the upper- and middle-third of the oesophagus to that observed in the lower-third (Table 1). While misclassification in the lower-third might well have caused a diagnostic shift from AGC to ACO, this would obviously have been extremely unlikely for the upper- and middle-third. Since a diagnostic shift limited to females is unlikely, the absence of these trends in females hardly weighs against this argument.

(2) A diagnostic shift of a size sufficient to explain the observed downward period effect in AGC incidence should have been reflected in a complementary upward period effect in ACO, which, however, was not observed (Figure 2). We therefore consider both the rising ACO incidence and the declining AGC incidence to be genuine.

There was no significant nonlinear male ACO cohort effect, meaning that the annual changes in ACO incidence did not differ significantly by year of birth (Table 3). However, the annual increases were considerable, amounting to over 9% in the youngest age cohorts. In the absence of factors causing a period effect, such as a major change in tumour classification or the appearance of a new carcinogen, and because of the presence of a cohort effect in females, we are convinced of a cohort effect in males with ACO, thus confirming the cohort effect for ACO found in US SEER-data (El-Serag *et al*, 2002).

The two-fold gender differences in the annual percentage increases in ACO incidence (Table 1) were surprising, as the annual incidence increase in Barrett's oesophagus in the Netherlands was recently demonstrated to be equal for both genders (van Soest *et al*, 2005). However, the source of this gender difference was revealed to be a steep dip in the annual increase in ACO incidence for females, and to a far smaller extent in males, aged 61–84, that is those born between 1905 and 1940 (Table 2). This dip was not a temporary phenomenon as the increases at ages 40–85+ were calculated separately for each of the 15 years. We suggest nonsteroidal anti-inflammatory drug (NSAID) use by the elderly as a possible cause of this dip. Several studies have suggested a protective effect of NSAIDs against ACO in patients with Barrett's oesophagus (Souza *et al*, 2000; Buttar *et al*, 2002; Kaur *et al*, 2002; Bardou *et al*, 2004; Gammon *et al*, 2004; Tsibouris *et al*, 2004; Vaughan *et al*, 2005). In addition, there are marked gender differences in NSAID use in the elderly (Schnitzer *et al*, 2001; Helin-Salmivaara *et al*, 2003). In the Netherlands, the Integrated Primary Care Information electronic database covering 500 000 patients revealed the NSAID user rate, rising from 10% below 45 to 23% over 45 years and a 1.5-fold higher user rate in females (Dr MCJM Sturkenboom, personal communication). In individuals over the age of 61 with Barrett's oesophagus, NSAID use, by postponing or preventing the onset of ACO, is likely to have contributed to the reduction in the annual rise of ACO incidence, this effect being more marked in females, consistent with their higher NSAID consumption.

The declining AGC incidence raises questions about the nature of AGC and its aetiology. The most commonly used Siewert classification defines AGC as an adenocarcinoma with its centre 5 cm proximal or distal from the anatomical cardia and distinguishes it into three types. Type I arising from (short segment) Barrett's oesophagus and therefore, in our opinion, ACO; type II, true AGC, arising from the cardiac epithelium or a short segment of intestinal metaplasia at the oesophagogastric junction; and type III, subcardial gastric cancer, infiltrating the cardia from below (Siewert and Stein, 1998). Since it has been suggested that there are two types, one related to *Helicobacter pylori* and the other to gastro-oesophageal reflux (McColl, 2006), AGC may well reflect the sum of these two opposing aetiologies.

The suspected causes of the increasing ACO incidence in industrialised countries, increasing obesity and possibly the declining prevalence of *H pylori* infection, are both evident in the Netherlands (Roosendaal *et al*, 1997; Richter *et al*, 1998; Visscher *et al*, 2002; El-Serag *et al*, 2005). Mediating their effects through reflux oesophagitis and Barrett's oesophagus (Souza and Spechler, 2005), these factors appear less relevant for AGC, as demonstrated by a recent meta-analysis of overweight as a risk factor for gastro-oesophageal reflux disease, which, in marked contrast to ACO, found only a marginally increased risk of AGC from obesity (Hampel *et al*, 2005). Studies of *H pylori* infection in AGC also failed to find a significant relationship (Hansen *et al*, 1999; Wu *et al*, 2003; Gulmann *et al.*, 2004).

Smoking, however, may well be important in the aetiology of AGC. In a prospective study with accurate differentiation between ACO and AGC, AGC was found to be dose-dependently related to smoking, odds ratio 4.2, whereas the relation with ACO was weak or absent (Lagergren *et al*, 2000). A complementary observation was made in institutionalised intellectually disabled individuals who do not smoke (van Blankenstein *et al*, 2004).

**Figure 3:**
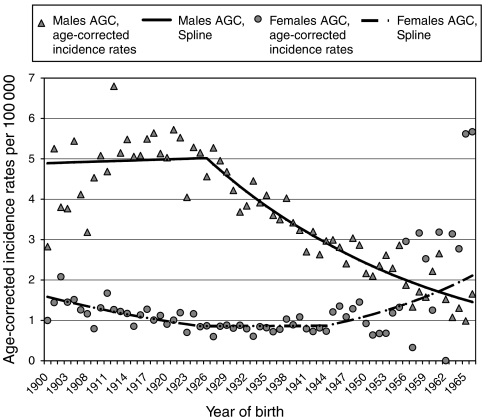
The age-cohort model diagram for AGC in males and females.

The contrasting AGC cohort effects in males and females (Figure 3) nicely fit the contrasting secular changes in smoking patterns in Dutch men and women, as implied by a lung cancer study, which found a steady decline in the number of male smokers born after 1914. This is in contrast to females, in whom, since the second half of the 19th century and in successive birth cohorts, the number of smokers has steadily increased and, after a relative plateau between 1928-37, has continued to increase, with numbers doubling in 1945 and later birth cohorts (Barendregt *et al*, 2002). The net decline in the AGC incidence is explained by the 4:1 male/female ratio, with the impact of the declining numbers of male smokers considerably outweighing that of the rising number of their female counterparts. We suggest that these findings lend support to a significant role for smoking in the aetiology of AGC.

Finally, the conversion of nitrite in saliva by acid into potentially mutagenic substances such as nitrous acid, nitrosative species and nitric oxide comprises a less well-established aetiology for the oesophagus–cardia adenocarcinoma which may have gained importance by the greater use of nitrogenous fertilizers after World War II (Spechler, 2002). In patients with Barrett's oesophagus, mutagenic nitrite conversion has recently been shown to occur within the Barrett's oesophagus segments (Suzuki *et al*, 2005). For AGC, this conversion is therefore likely to be localised in the cardia (McColl, 2005). Whether this hypothetical factor is still on the increase or has passed its peak is unknown.

## Figures and Tables

**Table 1:** Per annum percentage changes in the incidence rates of adenocarcinoma of the oesophagus, overall and by level, and of the cardia, 1989–2003, by gender

**Oesophageal tumour**	**Gender**	**Numbers observed**	**Incidence ESR**	**Annual %change**	**95% CI annual change**	***P*-values**
ACO	Males	4949	4.43	+7.2	+6.5; +7.9	<0.001
Overall	Females	1589	0.96	+3.5	+2.3; +4.7	<0.001
ACO	Males	82	0.07	+8.9	+2.6; +14.0	0.004
Upper-third	Females	77	0.04	+1.3	−3.8; +6.8	0.6
ACO	Males	340	0.3	+6.5	+3.9; +9.3	<0.001
Middle-third	Females	302	0.17	+1.0	−1.6; +3.7	0.5
ACO	Males	4220	3.8	+8.8	+8.1; +9.6	<0.001
Lower-third	Females	1059	0.7	+6.1	+4.6; +7.6	<0.001
ACO	Males	307	0.28	+1.5	−1.1; +4.2	0.3
Unspecified	Females	151	0.1	−0.4	−4.1; +3.3	0.8
AGC	Males	4863	4.34	−1.7	−2.4; −1.1	0.0002
Overall	Females	1441	0.92	−1.2	−2.4; −0.0	0.05

ACO=adenocarcinoma of the oesophagus; AGC=adenocarcinoma of the gastric cardia; CI, confidence interval; ESR=European standardised rates. Source incidence data: The Netherlands Cancer Registry.

**Table 2:** The per annum percentage increase in the incidence rates of adenocarcinoma of the oesophagus by age and gender

**Age bands**	**40–60**	**61–66**	**67–72**	**73–78**	**79–84**	**85+**
Males	9.0	5.9	6.0	7.0	7.8	5.5
Females	7.5	4.8	3.6	0.6	2.5	4.3

**Table 3:** Cohort or period effects for adenocarcinoma of the oesophagus (ACO) and the gastric cardia (AGC) by gender 1989–2003

			**Cohort effect**				**Period effect**			
**Tumour**	**Gender**	**Drift**	**<1926**	**1926–44**	**>1944**	***P***-**value**	**1989–93**	**1994–99**	**1999–03**	***P*-value**
ACO	Males	+7.2	+7.2	+7.1	+9.1	0.08	+6.8	+6.5	+8.4	0.5
ACO	Females	+3.5	+2.1	+7.1	+4.0	0.006	+2.9	+2.9	+ 5.4	0.6
AGC	Males	−1.7	+0.1	−3.0	−3.1	<0.0002	+1.4	−1.2	−5.9	0.0001
AGC	Females	−1.2	−2.6	+0.1	+4.1	0.01	+1.4	−1.4	−3.4	0.3

Drift=per annum percentage change.
